# Policy dialogue on health technology assessment in Middle East and North Africa: reporting from an HTAi initiative

**DOI:** 10.1017/S0266462326103870

**Published:** 2026-06-18

**Authors:** Mouna Jameleddine, Antonio Migliore, Ann Single, Rabia Sucu, Debjani Mueller, Nicola Vicari, Maitha Musabeh Ali Khalifa Alghfeli, Bader Alkhurainej, Emad Almomani, Hajer Al Mudaihem, Kefah Ali Al Alqawasmeh, Mouza Al Saadi, Anas Hamad, Zakia Ines Harzallah, Nabil Harzallah, Rita Karam, Kamel Mansouri, Said Wani, Amal Yassine, Awad Mataria, George Valiotis

**Affiliations:** 1 National Authority for Assessment and Accreditation in Healthcare (INEAS), Tunisia; 2 Health Technology Assessment International, HTAi, Canada; 3 Abu Dhabi Department of Health, United Arab Emirates; 4 Kuwait Pharmaceutical Association (KuPhA), Kuwait; 5 Maastricht University, Netherlands; 6 Saudi Society of Clinical Pharmacy, Saudi Arabia; 7 Dubai Health Authority, United Arab Emirates; 8 Ministry of Public Health Qatar, Qatar; 9 Faculty of Sciences and Medical Sciences, Lebanese University, Lebanon; 10 Algerian Society of Health Policies and Economics, Algeria; 11 Directorate General of Medical Supply, Government of Oman Ministry of Health, Oman; 12 Moroccan Society for Health Products Economics, Morocco; 13 EMRO, Egypt

**Keywords:** health technology assessment, Middle East and North Africa, policy dialogue, collaboration, institutionalization, capacity building, governance

## Abstract

**Objectives:**

Health technology assessment (HTA) is a multidisciplinary, policy-oriented process designed to inform evidence-based, transparent, and value-based decision making in health systems. Across the Middle East and North Africa (MENA), HTA development remains heterogeneous and unevenly institutionalized. This manuscript reports on a Health Technology Assessment international (HTAi)-led Policy Dialogue designed to identify shared challenges, priorities, and opportunities to strengthen HTA systems across the region.

**Methods:**

Insights were generated through an in-person meeting held in Tunisia in 2025, alongside the HTAi MENA Regional Meeting. The dialogue was complemented by an online survey and a follow-up virtual meeting. Data sources included structured survey responses from experts across twelve countries and qualitative inputs from multi-stakeholder discussions involving policymakers, HTA practitioners, academics, and international organization representatives. Findings were synthesized descriptively to capture perceived challenges, priorities, and expectations for regional and international collaboration.

**Results:**

Participants emphasized HTA’s role in improving resource allocation, transparency, and access to high-value technologies. Key challenges included limited access to high-quality data, workforce capacity constraints, fragmented governance, and weak legal and institutional frameworks. The highest-ranked priorities were establishing formal legal mandates for all actors involved in HTA processes, building technical and system capacity (including institutional governance, processes, and coordination mechanisms), and strengthening health data infrastructure. Strong support emerged for regional cooperation through joint training, shared assessments, and HTA networking, alongside expectations for international organizations to play a catalytic role.

**Conclusions:**

HTA is gaining prominence across MENA, but most countries remain in early phases of institutional development. Progress will require coordinated action on governance, capacity, data systems, and implementation.

## Background

The Middle East and North Africa (MENA) region comprises, in general, twenty countries extending from Morocco to Iran (there is no standardized definition and groupings may vary), accounting for almost 6 percent of the world’s population. Health systems across MENA face complex challenges, including rising healthcare costs and an increasing burden of chronic diseases combined with increasing expectations from the population. Rapid demographic and epidemiological transitions have amplified needs for effective, evidence-based decision making to ensure resource efficiency and equity, especially in consideration of the increasing demand for costly innovative technologies (1). As a result, there is a growing recognition of the importance of health technology assessment (HTA) in the MENA region as a critical tool for evidence-based decision making in health care (2), particularly in contexts where health systems face increasing cost pressures, competing priorities, and the need to optimize the allocation of limited resources (3), although many countries still rely on unstructured or *ad hoc* evaluation processes (4). As part of an ongoing, member-led effort to strengthen HTA practice across MENA (5), Health Technology Assessment international (HTAi) supported a Policy Dialogue hosted in Tunis, Tunisia, on 24 September 2025, alongside its MENA Regional Meeting (5). The Dialogue was co-developed with local HTAi members and regional stakeholders to ensure that discussions, priorities, and proposed actions were addressing gaps in HTA implementation, grounded in local realities, and led by regional expertise. The present manuscript reports on an HTAi-led Policy Dialogue in the MENA region, synthesizing participants’ discussions and survey findings to identify common challenges, priority areas, and opportunities to strengthen HTA systems at national and regional levels.

## Policy Dialogue meeting structure and methods

HTAi Policy Dialogues are intended to underscore the significance of collaboration and co-creation among the stakeholders of various countries, providing a “safe space” that encourages participants to discuss and subsequently collaborate to create sustainable HTA frameworks that can be customized to the specific needs of their respective countries (6). HTAi Policy Dialogues require participants to engage openly and constructively, expressing views and sharing ideas independently of their formal institutional roles or affiliations. This approach aims to foster candid exchange, mutual understanding, and collaborative exploration of shared challenges and solutions to ensure that HTA is a fully functional and integral component of their healthcare decision making.

### In-person meeting (24 September 2025)

The HTAi Policy Dialogue for MENA began with a structured, in-person meeting held alongside the HTAi MENA Regional Meeting in Tunis, Tunisia. Twenty participants from twelve countries (ten MENA countries plus Australia and South Africa) took part. The meeting followed a facilitated roundtable format, combining plenary discussion and guided exchanges to identify shared challenges in HTA development, priority areas for system strengthening, and expectations for regional and international collaboration. Discussions were informed by short introductory inputs and were documented through moderator notes to support subsequent analysis.

### Online survey (1 October 2025–4 December 2025)

Following the in-person meeting, participants co-developed and refined an online survey designed to systematically capture and prioritize perceived challenges, drivers, and strategic needs for HTA development across national contexts. The survey included structured ranking questions and open-ended items (see Supplementary Material). Participants were recruited through convenience sampling, drawing primarily from professionals already identified by the Organizing Committee of the HTAi Regional Meeting for MENA and invited to present at the event. To broaden geographical reach and professional representation, those participants were invited to share the survey link within their professional networks from their respective countries, particularly individuals working in HTA agencies and other entities conducting HTA or HTA-like activities, such as ministries of health, academia, or related institutions (open, network-based dissemination approach). Stakeholder affiliation categories were defined pragmatically to reflect institutional roles relevant to HTA policy development and system implementation.

### Virtual meeting and validation (18 December 2025)

A virtual follow-up meeting was convened to discuss and validate the draft survey findings. Participation in this meeting was open to all individuals who had taken part in the in-person Policy Dialogue. The meeting focused on interpreting survey results, confirming their consistency with regional experience, and contextualizing the findings for inclusion in the manuscript.

The survey results, integrated with the in-person and virtual discussions, were used to capture and prioritize perceived challenges, strategic needs, and expectations for HTA development and regional collaboration, forming the empirical basis for the present analysis. All co-authors of this manuscript were involved in at least one of the Policy Dialogue activities (in-person meeting and/or virtual discussion) and contributed to the interpretation of findings and manuscript development.

## Results from the online survey

### Participants and their characteristics

Responses, fifty-two in total, were received from participants from twelve countries: Algeria (*n* = 3), Egypt (*n* = 3), Iran (*n* = 1), Jordan (*n* = 3), Kuwait (*n* = 1), Lebanon (*n* = 8), Morocco (*n* = 2), Oman (*n* = 9), Qatar (*n* = 4), Saudi Arabia (*n* = 6), Tunisia (*n* = 5), and United Arab Emirates (*n* = 6). One response was received from an inter-governmental organization, World Health Organization (WHO) EMRO. In terms of affiliation, most of the respondents were working for governmental institutions, such as the Ministry of Health or HTA bodies (twenty-eight out of fifty-two; 54 percent), while others were academics (8/52; 15 percent) or worked at payer or insurer bodies (five out of fifty-two; 10 percent). Industry, patients’ organizations, and healthcare providers were less represented (overall, seven out of fifty-two; 13 percent). Others included consulting and non-governmental organizations (four out of fifty-two; 8 percent). All but four respondents indicated previous involvement in HTA. Most of the respondents (thirty-seven out of fifty-two; 71 percent) disclosed their role. Among them, the largest group (fifteen out of thirty-seven; 41 percent) described themselves as directors, leads, coordinators, or heads of departments, reflecting leadership or managerial roles in national or institutional HTA activities. Others (eleven out of thirty-seven; 30 percent) identified themselves as committee members. Six respondents reported to have a technical role (six out of thirty-seven; 16 percent), whereas five classified themselves as health economists (five out of thirty-seven; 13 percent). The vast majority of respondents (forty out of fifty-two; 77 percent) disclosed their involvement in HTA in terms of years, showing a seasoned cohort, combining mid-career and senior experts: ten out of forty (25 percent) were senior-level professionals with ten or more years of engagement in HTA; fifteen out of forty (37 percent) reported between 4 and 10 years of involvement, typically mid-career experts with solid experience; another substantial group, eleven out of forty (28 percent) comprised early-career respondents with fewer years of experience. Only four respondents (four out of forty; 10 percent) indicated to be completely new to the field, with 1 year or less of experience.

### HTA institutionalization

Survey participants were asked to describe the level of institutionalization of HTA in their countries together with the technologies targeted by HTA activities (e.g., pharmaceuticals, medical devices, public health interventions, digital health solutions) and the use of HTA output in decision making (e.g., informal advice, formal appraisal). Since definitions of institutionalization and use were not included in the questions, responses varied largely and presented conflictual interpretations for some countries. These questions were excluded from the analysis and only the HTA scenario in each country was presented, combining free searches and insights from the authoring group. Discussions among the authoring group consolidated the assumption that institutionalization is strongly related to the existence of an independent HTA body or any HTA or HTA-like function formally embedded within ministries or governmental institutions. Table 1 provides a descriptive overview of the HTA landscape across the MENA region, highlighting selected milestones and planned developments without implying comparison among countries. The information suggests that several countries are engaged in efforts to strengthen or formalize HTA-related functions, reflecting diverse national contexts and priorities.

**Table 1.** Status of health technology assessment (HTA) across Middle East and North Africa (MENA) Table 1. long description.

Country	HTA Status	Notes
Algeria (7)	In progress	No official, independent HTA agency at the national level that performs systematic HTA as part of pricing and reimbursement decisions, whereas there are directorates across several ministries responsible for economic evaluation, pricing, and reimbursement, respectively.
Egypt (8)	In progress	The national Unified Procurement Authority, responsible for centralized procurement and supply of health technologies, conducts evidence-based HTA to support decision making for procurement and resource allocation. The World Health Organization has supported training programs to strengthen local skills in cost-effectiveness analysis and other HTA domains. The Ministry of Health and Population is planning to advance a multidisciplinary National HTA Framework.
Iran (9)	Incomplete	Formally present since the late 2000s with an HTA office within the Ministry of Health and Medical Education; while systematic evaluations of health technologies are produced, HTA is not yet fully integrated into national health policy or decision making due to limitations in legal frameworks, funding, capacity, and report quality.
Jordan (10)	Institutionalized	Within the Royal Medical Services, a government-affiliated healthcare organization, an independent HTA unit is responsible for producing HTA reports and is active since 2020. Formal HTA function within the Ministry of Health’s Health Economics Department began in 2023 with the establishment of national HTA committees. National HTA guidelines were developed in 2025.
Kuwait (11)	In progress	In November 2025, the Minister of Health officially approved the national Roadmap for HTA framework. In December 2025, the Ministry of Health officially updated its organizational chart to establish a formal HTA structure with specific departments for reimbursement and for pricing. Publication of Kuwait’s first Multiple Criteria Decision Analysis framework, which incorporates a set of criteria specifically for genetic medications, is ongoing.
Lebanon (12)	In progress	HTA is not formally institutionalized. In January 2025, the first national economic evaluation guideline (the Lebanese Economic Evaluation Guideline) was released.
Morocco (13)	In progress	The High Authority for Health was established under the reform of the healthcare system (2023). The new body, not yet operational, has the mission of evaluating healthcare services and products and will be responsible for implementing HTA. In March 2025, the first national EQ–5D–5L value set was published, a crucial tool for calculating Quality-Adjusted Life Years in economic evaluations within HTA. The first University Diploma in HTA was implemented through a partnership between academia, scientific societies, the Ministry of Health and Social Protection, and industry partners.
Oman (14)	In progress	In 2024–2025, a roadmap was defined to institutionalize HTA following a phased approach (10-year plan), which started with building capacity through advanced training programs, developing the first national HTA methodological guidelines, establishing a national HTA unit housed within the Ministry of Health and applying HTA progressively to medical devices and surgical interventions after pharmaceuticals.
Qatar (15)	In progress	In January 2025, the Ministry of Public Health formally launched a dedicated project to establish a National HTA Unit, under the umbrella of Qatar National Health Strategy 2024–2030. A national HTA framework, complete with methodological standards for evaluating the clinical, economic, social, and system-level impacts of new health technologies, is currently under development. Multiple high-level training workshops for policymakers and technical experts have been delivered in collaboration with academic institutions and private partners. More capacity-building efforts are in the plan.
Saudi Arabia (16)	In progress	The wider healthcare system is undergoing large-scale transformation: the Ministry of Health is shifting from direct service provision to a role focused on policy and regulation, paving the way for a more privatized and efficient system. HTA has recently been formalized with the Centre of HTA responsible for the economic evaluation of pharmaceutical products to support reimbursement decisions across public and private sectors. While the formal process has been introduced, full implementation is expected over the coming years. The internal structure of CHTA is still evolving.
Tunisia (17)	Institutionalized	The National Authority for Assessment and Accreditation in Healthcare is the independent, national authority funded by the government and established in 2012 with HTA function from 2018. Other than assessing the added benefit and cost-effectiveness of health technologies and providing rigorous evidence-based recommendations to decision makers on pharmaceuticals and other technologies’ uptake and use, INEAS responsibilities include accreditation of healthcare organizations and production of clinical practice guidelines and care pathways. INEAS is the only INAHTA member from MENA.
United Arab Emirates (18;19)	Institutionalized^a^	In 2025, the Emirates Health Services has created a phased HTA framework. In June 2025, the Department of Health of Abu Dhabi published official HTA guidelines and started implementation.

a Implemented only within the Emirate of Abu Dhabi.

### Drivers and challenges in the implementation of HTA

Respondents were asked regarding their primary reason for implementing or supporting the implementation of HTA in their jurisdiction. They broadly agreed that the primary motivation was to strengthen evidence-based, transparent, and equitable decision making. A key theme was optimizing resource allocation in constrained health budgets by identifying high-value interventions, reducing inefficiencies, and improving value for money. Improving access to effective and innovative technologies was another central motivation. HTA was viewed as a tool to guide the introduction of new medicines, devices, digital health solutions, and emerging therapies, ensuring that these deliver meaningful benefits relative to cost. Participants also emphasized HTA’s role in enhancing transparency and consistency in policy and reimbursement decisions through standardized evaluation processes, regulatory alignment, and structured priority setting. Finally, several respondents highlighted HTA’s contribution to clinical and system improvement, emphasizing its relevance for strengthening national health strategies, aligning with global best practices and WHO recommendations, and positioning their country as a regional leader in value-based health care. Other motivations were more context-specific but aligned with broader themes, for example, managing oversubscription of new medicines in tendering systems, supporting managed entry agreements, tailoring interventions to population needs, and improving coverage decisions in public insurance/prepayment schemes.

**Figure 1.**
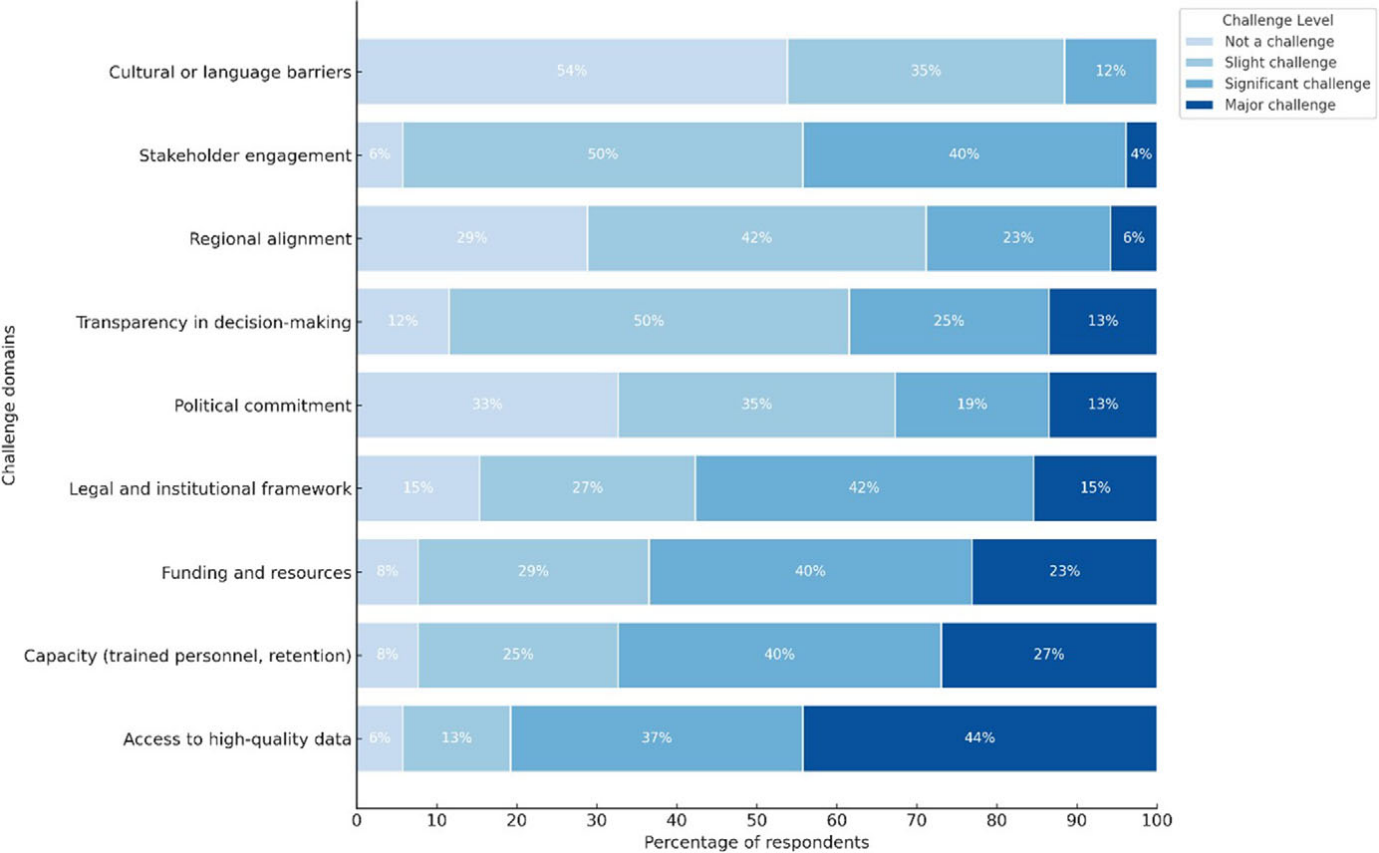
Distribution of how respondents (*n* = 52) perceived challenges across health technology assessment–related domains. Figure 1. long description.

Respondents were asked to rate the level of challenge in their context across nine dimensions (Figure 1). The quantitative findings were reinforced by open-ended comments that provided additional detail and contextual nuance. The most significant challenge, reported by over two-thirds of respondents, concerned access to high-quality data. Experts cited fragmented information systems, limited local epidemiological and economic data, and concerns about the reliability of externally sourced evidence, particularly in the context of rapid or expedited assessments. Workforce capacity, funding and resources, and the legal and institutional framework were rated as major or significant challenges by more than half of respondents. Participants highlighted workforce shortages and retention difficulties, including the loss of trained HTA experts and insufficient specialist capacity. Governance and political instability emerged as cross-cutting issues affecting multiple domains. The respondents perceived that the effectiveness of political commitment and institutional frameworks was undermined by frequent leadership changes, limited continuity of reforms, and unclear decision-making processes. Institutional fragmentation also emerged as a recurrent theme. Commenters reported overlapping responsibilities among agencies, weak alignment between HTA outputs and reimbursement or procurement decisions, and duplication of efforts. While other dimensions, such as political commitment, transparency in decision making, and regional alignment, were rated as not challenging or only slightly challenging by over two-thirds of respondents, a minority still reported these areas as major challenges. In contrast, stakeholder engagement was rated between significant and slightly challenging by nearly all respondents. Cultural or language barriers were not considered a challenge at all by over half of respondents. Additional remarks highlighted emerging or context-specific issues, including the adaptation of HTA recommendations to local value-based purchasing models, the management of rapidly evolving technologies (e.g., in oncology), inconsistencies between public- and private-sector implementation, and the need for stronger multidisciplinary collaboration.

### Priorities for strengthening HTA development

**Figure 2.**
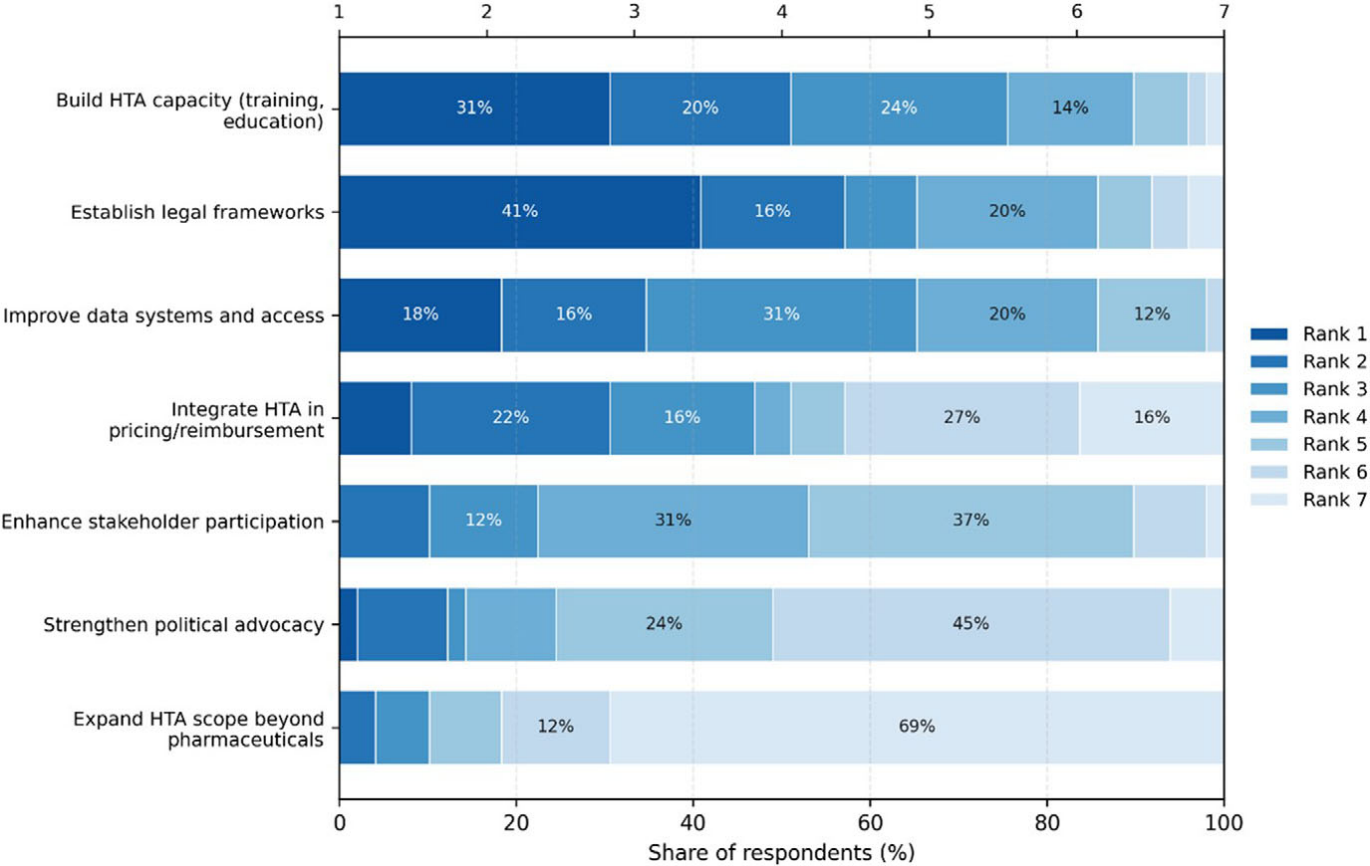
How the respondents (*n* = 49) ranked the proposed priorities for their contexts (Rank 1: highest priority). Segments <10% are not labeled in the graph. Figure 2. long description.

Experts were asked to select and rank their top three priorities for strengthening HTA development in their countries. Clear priority areas emerged from the responses (Figure 2). The priorities that were ranked highest (rank 1) more often were *Establishing legal frameworks* (twenty respondents), followed by *Building HTA capacity* (fifteen respondents) and *Improving data systems and access* (nine respondents).

When asked about which urgent actions should be taken in the next 2 years, respondents identified a clear and ambitious agenda for strengthening HTA systems. The most frequently cited priority was the establishment or finalization of a national HTA legal and governance framework (including ministerial decisions, formal HTA entities, clearly defined roles and responsibilities across government stakeholders, and standardized processes for topic selection, submission, appraisal, and decision making). Another central theme was the need to upgrade national data infrastructures to support HTA. Respondents called for improved access to high-quality clinical, economic, and real-world data; development of national registries; digitalization of health systems; and clear data-sharing agreements. Several stressed that data systems must be built on strong legal and methodological foundations, and that implementing registries without proper frameworks risks wasting already limited resources.

Capacity building emerged as an equally urgent priority. Many respondents highlighted the shortage of trained HTA professionals, including economists and analysts, calling for postgraduate specialization, training programs, short courses, and increased certification efforts. However, the lack of system capacity was also mentioned, in terms of training of HTA users such as policy makers and clinicians. Stakeholder engagement efforts were also seen as essential, both for developing a shared understanding of HTA and ensuring that clinicians, academia, patients, and industry are meaningfully involved early in the HTA process. Respondents also emphasized the importance of integrating HTA into decision-making processes, that is, embedding HTA into reimbursement, pricing, formulary inclusion, procurement, and priority-setting mechanisms. Several mentioned piloting HTAs for high-cost or high-impact innovations, developing early scientific advice pathways, and beginning to implement managed entry agreements or structured evaluation criteria.

Sustainable financing was another major concern. Some proposed manufacturer submission fees, whereas others emphasized the need for government-backed funding streams to support HTA units, local data systems, and ongoing capacity-building activities. In addition, many respondents called for the development or enforcement of national HTA guidelines, standardized evaluation methodologies, costing manuals, and topic selection scorecards. These tools were seen as essential for improving transparency, consistency, and quality across assessments. Finally, respondents stressed the need for greater coordination and communication, both within and across sectors. This includes unifying protocols among multiple public payers, mapping roles and responsibilities across institutions, ensuring alignment with broader health reforms, and strengthening regional collaboration, in particular to share expertise, reduce duplication, and harmonize approaches across the MENA region.

### Regional cooperation and international partnerships

Respondents indicated clear preference to specific forms of regional cooperation which were proposed in the survey question. The establishment of *Joint training programs* and a *Regional HTA network* were the initiatives that were most favorably received (forty-four out of fifty-two; 85 percent). However, also *Shared assessments* and *Data sharing platforms* were believed needed by most (thirty-three out of fifty-two; 63 percent and thirty-one out of fifty-two; 60 percent, respectively). Respondents identified a diverse set of international organizations they believe could play a meaningful role in strengthening HTA capacity within their countries. The most frequently mentioned institutions were those with a strong global footprint in HTA methodology, capacity building, and evidence-based policymaking. HTAi emerged as the most commonly cited organization (twenty-nine mentions), reflecting its global role as a professional society supporting HTA development, networking, and methodological advancement. The International Network of Agencies for Health Technology Assessment (INAHTA) followed closely (nineteen mentions), showing strong interest in peer-to-peer collaboration with established HTA agencies. The WHO was also frequently identified (sixteen mentions), highlighting the importance of international guidance, standard-setting, and technical support, especially in low- and middle-income settings. Other organizations such as the International Society for Pharmacoeconomics and Outcome Research and the National Institute for Health and Care Excellence were mentioned (fifteen and eleven times, respectively) in relation to the demand for methodological guidance, health economics expertise, and practice models. A smaller number of respondents identified organizations such as the Canada’s Drug Agency (four mentions), the Organization for Economic Co-operation and Development (three mentions), the International Health Economics Association, the *Haute autorité de santé*, and the Institute for Clinical and Economic Review (two mentions each), as well as regional bodies like the Gulf Health Council (one mention).

More specifically, respondents were asked how HTAi could strengthen HTA capacity, institutionalization, and regional collaboration, with a wide range of initiatives proposed. Qualitative analysis allowed to cluster them into five dominant themes. Capacity building was the most prevalent theme (thirty-two mentions) and included requests for foundational and advanced HTA training, short courses, postgraduate programs, certification pathways, and support for building local HTA units. Many respondents (twenty-seven mentions) called for HTAi to support countries in developing national data infrastructures, enabling access to real-world data, guiding methodological standards, and offering training in modern analytic tools, including artificial intelligence and advanced economic evaluation methods. Regional collaboration and networking were the third most frequently cited theme (twenty mentions). Respondents highlighted the value of HTAi facilitating cross-country learning, organizing regional meetings, strengthening MENA collaboration, and promoting joint activities among stakeholders who face similar HTA system challenges. A fourth cluster centered on stakeholder engagement (thirteen mentions). Experts expressed the need for HTAi to help build platforms that engage policymakers, clinicians, academia, patients, and industry in HTA discussions. Several noted that HTA uptake is more successful when stakeholders are involved early and consistently. Finally, respondents saw HTAi as a credible, neutral actor that could help countries formalize HTA processes and promote evidence-informed policymaking in a role of advocacy and policy support (twelve mentions).

## Discussion

The HTAi Policy Dialogue for MENA portrays a region-wide consensus about the fact that HTA is not merely a technical exercise, but a multidisciplinary, policy-oriented process designed to inform evidence-based, transparent, and value-based decision making in health systems. Respondents’ emphasis on resource optimization, structured decision rules, and improved access to effective innovations aligns with global evidence demonstrating that institutionalized HTA reduces unwarranted variation, strengthens priority setting, and enhances accountability in health spending. This is consistent with comparative studies across low- and middle-income countries, which identify similar drivers for HTA adoption, including fiscal constraints, rapid technology diffusion, and the need for consistent reimbursement processes (20;21).

Importantly, the findings distinguish between structural challenges and strategic priorities. Core system bottlenecks include the absence of integrated health and cost data platforms, limited technical and analytical workforce capacity, fragmented governance arrangements, and unclear institutional mandates. These constraints impede the routine production, appraisal, and uptake of HTA outputs. In response, countries articulated clear priorities: establishing formal legal and regulatory frameworks for HTA; delineating institutional roles and decision rights; standardizing processes for topic selection, evidence appraisal, stakeholder consultation, and policy implementation; and investing in interoperable data infrastructure and workforce development. This sequencing reflects international experience, where successful HTA institutionalization typically begins with governance consolidation and procedural formalization before progressing to more advanced functions such as early scientific advice and horizon scanning (22).

Across the above domains, sustainable financing and sustained advocacy emerge as cross-cutting enablers. Dedicated and predictable funding mechanisms, whether through earmarked public budgets, integration into payer financing streams, or hybrid models, are essential to ensure governance continuity, retain skilled personnel, and maintain methodological quality over time. Without stable financing, HTA units risk project-based operation, staff attrition, and diminished policy influence. Similarly, ongoing advocacy directed at political leaders, payers, clinicians, and patient groups is critical to reinforce the legitimacy of HTA, embed its use in routine decision making, and safeguard it from short-term political or fiscal fluctuations. Together, financing and advocacy underpin long-term system resilience and the durability of HTA institutions.

The expressed demand for regional cooperation, through joint training initiatives, shared assessments, and data-sharing platforms, further reinforces opportunities for collective action in MENA. Regional HTA collaboration has demonstrated value in the European Union, Latin America, and Asia-Pacific, where coordinated approaches have reduced duplication, improved methodological consistency, and accelerated evidence generation for shared policy questions (23;24). Comparable gains are plausible in the MENA region, where shared epidemiological profiles and procurement challenges intersect with growing pharmaceutical expenditure and expanding technology uptake across countries (25).

The strong expectation that HTAi could play a catalytic role in capacity building, methodological standardization, advocacy support, and stakeholder engagement suggests a clear mandate for international partnership. Respondents envision HTAi as a central partner in strengthening HTA across the region, not only through technical skill development but also through system-level support, consensus building, and fostering a cohesive regional HTA community. Collectively, these findings delineate a pragmatic roadmap for HTA institutionalization in MENA: addressing foundational challenges in governance and data systems; prioritizing formal mandates, procedural clarity, and workforce investment; securing sustainable financing and sustained advocacy; embedding HTA into routine policy processes; and leveraging regional and international collaboration to accelerate and sustain progress.

## Limitations

The present study has several limitations that should be acknowledged when interpreting the findings. The Policy Dialogues represent HTAi’s effort to support the development of HTA in specific regions of the world. The MENA region was targeted given that structural initiatives to connect the countries around HTA are not currently in place. Although the meeting was able to attract and involve several participants from different countries (Algeria, Egypt, Jordan, Kuwait, Lebanon, Morocco, Oman, Qatar, Saudi Arabia, Tunisia, and United Arab Emirates), it should be acknowledged that some countries were not represented at all during the in-person event such as Bahrain, Iraq, Iran, Palestine, either because invitations were not issued for capacity reasons or invited people were unable to join (Saudi Arabia). However, those who did not attend received the invitation to participate in the survey. According to the HTAi Policy Dialogue’s concept, participants were asked to express their opinions regardless of the official position of the institutions of their countries. While this created a free environment for open discussion, it may also have introduced a disconnect. In addition, the unequal number of survey responses across countries and stakeholders may have resulted in some perspectives being more prominently reflected than others. Because the online survey was disseminated through an open, network-based approach, it was not possible to determine the total number of individuals who received the invitation and, therefore, a formal response rate could not be calculated.

The present manuscript reflects respondents’ perceptions rather than objective indicators of HTA status and, therefore, should be interpreted as an exploratory snapshot of views of a group of engaged experts in the MENA region, not as a comprehensive or statistically generalizable assessment of the whole region. Although a large group of respondents had leadership roles within governmental bodies that are currently key players in the HTA institutionalization process, the perceptions reported reflect a specific point in time within each country and may evolve as HTA systems, policies, and institutional arrangements continue to develop across the region. The survey relied on a convenience sample drawn largely from existing formal and informal networks, which introduces selection bias toward individuals who are already engaged and generally favorable to HTA. The analysis was deliberately descriptive because differences in perceptions across countries, HTA maturity levels, or stakeholder types were not explored. Qualitative comments given by experts were used illustratively without a formal thematic analysis, so the depth of interpretation is limited. Nonetheless, the elements and insights generated through the present project offer a robust qualitative foundation for regional HTA strategy development.

## Conclusions and possible future steps

HTA is gaining strategic prominence across MENA, yet most countries remain in the early phases of institutional development. Strengthening HTA will require coordinated efforts to establish clear legal frameworks, invest in sustainable human capacity and data infrastructure, and ensure that assessment outputs inform reimbursement, pricing, and procurement decisions in a transparent and systematic manner. Regional cooperation, particularly joint training, shared assessments, and harmonized methodological guidance, offers a high-value opportunity to accelerate progress while reducing duplication of effort. Future steps may include creating a structured MENA HTA network, building on and learning from a previous attempt in this regard made by WHO EMRO, developing regional data standards, piloting cross-country collaborative assessments, and formalizing partnerships with organizations, such as HTAi, INAHTA, and WHO. These actions would support the maturation of HTA systems and promote more equitable, evidence-informed healthcare decision making across the region.

## Supporting information

10.1017/S0266462326103870.sm001 Jameleddine et al. supplementary material Jameleddine et al. supplementary material

## Supplementary material

The supplementary material for this article can be found at http://doi.org/10.1017/S0266462326103870.

## Long descriptions

### Table 1. Long description

The table consists of three columns: Country, H T A Status, and Notes.

* Algeria: In progress. No national agency; several ministries handle economic evaluation and pricing.

* Egypt: In progress. Unified Procurement Authority conducts evidence-based H T A; W H O supports training; Ministry of Health planning a national framework.

* Iran: Incomplete. H T A office exists since late 2000s but is not fully integrated into policy due to legal and funding limits.

* Jordan: Institutionalized. Independent unit in Royal Medical Services since 2020; Ministry of Health committees established in 2023; guidelines developed in 2025.

* Kuwait: In progress. Roadmap approved in November 2025; formal structure for reimbursement and pricing established in December 2025.

* Lebanon: In progress. Not formally institutionalized; first economic evaluation guidelines released in January 2025.

* Morocco: In progress. High Authority for Health established in 2023; E Q 5 D 5 L value set published in March 2025; first university diploma in H T A implemented.

* Oman: In progress. 10-year roadmap defined in 2024 to 2025 to build capacity and establish a national unit.

* Qatar: In progress. National H T A Unit project launched in January 2025 under the National Health Strategy 2024 to 2030.

* Saudi Arabia: In progress. Centre of H T A formalized for pharmaceutical economic evaluation; full implementation expected over coming years.

* Tunisia: Institutionalized. National Authority for Assessment and Accreditation in Healthcare established in 2012 with H T A function since 2018; only I N A H T A member in the region.

* United Arab Emirates: Institutionalized in Abu Dhabi. Emirates Health Services created a framework in 2025; Abu Dhabi published official guidelines in June 2025.

Navigate back to Table 1..

### Figure 1. Long description

The chart plots Challenge domains on the y-axis against Percentage of respondents on the x-axis, ranging from 0 to 100. A legend identifies four challenge levels: Not a challenge (lightest blue), Slight challenge, Significant challenge, and Major challenge (darkest blue).

* Cultural or language barriers: 54 percent Not a challenge, 35 percent Slight challenge, 12 percent Significant challenge.

* Stakeholder engagement: 6 percent Not a challenge, 50 percent Slight challenge, 40 percent Significant challenge, 4 percent Major challenge.

* Regional alignment: 29 percent Not a challenge, 42 percent Slight challenge, 23 percent Significant challenge, 6 percent Major challenge.

* Transparency in decision-making: 12 percent Not a challenge, 50 percent Slight challenge, 25 percent Significant challenge, 13 percent Major challenge.

* Political commitment: 33 percent Not a challenge, 35 percent Slight challenge, 19 percent Significant challenge, 13 percent Major challenge.

* Legal and institutional framework: 15 percent Not a challenge, 27 percent Slight challenge, 42 percent Significant challenge, 15 percent Major challenge.

* Funding and resources: 8 percent Not a challenge, 29 percent Slight challenge, 40 percent Significant challenge, 23 percent Major challenge.

* Capacity (trained personnel, retention): 8 percent Not a challenge, 25 percent Slight challenge, 40 percent Significant challenge, 27 percent Major challenge.

* Access to high-quality data: 6 percent Not a challenge, 13 percent Slight challenge, 37 percent Significant challenge, 44 percent Major challenge.

Navigate back to Figure 1..

### Figure 2. Long description

The chart features a horizontal axis representing the share of respondents from 0 to 100 percent and a top axis showing a rank scale from 1 to 7. A legend on the right indicates a color gradient from dark blue for Rank 1 to very light blue for Rank 7.

* Build H T A capacity training, education: Rank 1 at 31 percent, Rank 2 at 20 percent, Rank 3 at 24 percent, Rank 4 at 14 percent.

* Establish legal frameworks: Rank 1 at 41 percent, Rank 2 at 16 percent, Rank 3 at 20 percent.

* Improve data systems and access: Rank 1 at 18 percent, Rank 2 at 16 percent, Rank 3 at 31 percent, Rank 4 at 20 percent, Rank 5 at 12 percent.

* Integrate H T A in pricing or reimbursement: Rank 2 at 22 percent, Rank 3 at 16 percent, Rank 5 at 27 percent, Rank 6 at 16 percent.

* Enhance stakeholder participation: Rank 2 at 12 percent, Rank 3 at 31 percent, Rank 4 at 37 percent.

* Strengthen political advocacy: Rank 4 at 24 percent, Rank 5 at 45 percent.

* Expand H T A scope beyond pharmaceuticals: Rank 4 at 12 percent, Rank 5 at 69 percent.

Segments representing less than 10 percent of the share are visible but not labeled with numerical values.

Navigate back to Figure 2..
