# Supplementary material for: Policy dialogue on health technology assessment in Middle East and North Africa: reporting from an HTAi initiative
Source: Int J Technol Assess Health Care. 2026 Jun 18;42(1):e62. doi: 10.1017/S0266462326103870 (PMC13440439; doi:10.1017/S0266462326103870)
Supplement: Supplementary file 1 [file S0266462326103870sup001.pdf]

Challenges and Priorities to Advance HTA in MENA

Défis et priorités pour faire progresser l'évaluation des technologies de la santé (ETS) dans la région MENA

* Required

1. **Country / Pays** *

2. **Organization type / Type d'organisation** *

- ◯ Government / Gouvernement
- ◯ HTA Agency / Agence d'ETS
- ◯ Academia / Université, recherche
- ◯ Healthcare provider / Fournisseur de soins
- ◯ Payer or insurer / Payeur ou assureur
- ◯ Patient organization / Organisation de patients
- ◯ Industry / Industrie
- ◯ Other / Autre

3. If you replied "Other", please specify / Si "Autre", veuillez préciser

4. **Have you previously been involved in HTA activities? / Avez-vous déjà participé à des activités d'ETS?** *

- ◯ Yes / Oui
- ◯ No / Non

5. **Your role and years of experience in HTA or related fields / Votre rôle et années d'expérience en ETS ou domaines connexes** *

6. **Is HTA formally institutionalized in your country? / L'ETS est-elle institutionnalisée dans votre pays?**

- ◯ Yes / Oui
- ◯ No / Non
- ◯ In progress / En cours
- ◯ Not sure / Ne sait pas

7. **Primary focus areas for HTA in your country / Domaines prioritaires de l'ETS dans votre pays**

- ☐ Pharmaceuticals / Médicaments
- ☐ Medical devices / Dispositifs médicaux
- ☐ Public health interventions / Interventions de santé publique
- ☐ Digital health / Santé numérique
- ☐ Other / Autre

8. If you replied "Other", please specify / Si "Autre", veuillez préciser

9. **How are HTA outputs currently used in decision-making? / Comment les résultats de l'ETS sont-ils actuellement utilisés dans la prise de décision?**

- ☐ Informal advice / Avis informel
- ☐ Formal appraisal / Évaluation formelle
- ☐ Legally mandated / Obligation légale
- ☐ Not used / Non utilisé

10. **For each of the following, please rate the level of challenge in your context / Pour chacun des éléments suivants, veuillez évaluer le niveau de défi dans votre contexte**

	Not a challenge / Pas un défi	Slight challenge / Léger défi	Significant challenge / Défi important	Major challenge / Défi majeur
Political commitment / Engagement politique	○	○	○	○
Legal and institutional framework / Cadre juridique et institutionnel	○	○	○	○
Capacity (trained personnel, retention) / Capacité (personnel formé, rétention)	○	○	○	○
Access to high-quality data / Accès à des données de qualité	○	○	○	○
Stakeholder engagement / Implication des parties prenantes	○	○	○	○
Transparency in decision-making / Transparence dans la prise de décision	○	○	○	○
Funding and resources / Financement et ressources	○	○	○	○
Cultural or language barriers / Barrières culturelles ou linguistiques	○	○	○	○
Regional alignment / Alignement régional	○	○	○	○

11. **Other challenges (please specify) / Autres défis (veuillez préciser)**

12. **What is your primary reason for implementing - or supporting implementing - HTA in your jurisdiction? / Quelle est votre principale raison de mettre en œuvre – ou de soutenir la mise en œuvre – de l'ETS dans votre juridiction ?**

13. **Select and rank your top 3 priorities for HTA development in your country / Sélectionnez et classez vos 3 principales priorités pour le développement de l'ETS dans votre pays**

- Establish legal frameworks / Mettre en place des cadres juridiques
- Build HTA capacity (training, education) / Renforcer les capacités en ETS (formation, éducation)
- Improve data systems and access / Améliorer les systèmes de données et l'accès
- Enhance stakeholder participation / Renforcer la participation des parties prenantes
- Strengthen political advocacy / Renforcer le plaidoyer politique
- Integrate HTA in pricing/reimbursement / Intégrer l'ETS dans la tarification/le remboursement
- Expand HTA scope beyond pharmaceuticals / Élargir le champ de l'ETS au-delà des médicaments

14. **What urgent actions should be taken in the next 2 years? / Quelles actions urgentes devraient être entreprises dans les 2 prochaines années ?**

15. **Preferred forms of regional cooperation / Formes de coopération régionale préférées**

- ☐ Shared assessments / Évaluations partagées
- ☐ Joint training programs / Programmes de formation conjoints
- ☐ Data sharing platforms / Plateformes de partage de données
- ☐ Regional HTA network / Réseau régional d'ETS
- ☐ Other / Autre

16. If you replied "Other", please specify / Si "Autre", veuillez préciser

17. **Which international organizations do you believe could support your country in advancing HTA? / Quelles organisations internationales pourraient, selon vous, aider votre pays à faire progresser l'ETS ?**

18. **What initiatives should HTAi undertake to advance HTA in your region? / Quelles initiatives HTAi devrait-elle entreprendre pour favoriser l'avancement de l'ETS dans votre région ?**

19. **Preferred language(s) for training and resources / Langue(s) préférée(s) pour la formation et les ressources**

- [ ] Arabic / Arabe
- [ ] English / Anglais
- [ ] French / Français
- [ ] Bilingual / Bilingue

This content is neither created nor endorsed by Microsoft. The data you submit will be sent to the form owner.

Microsoft Forms
